# Ammonia and the Muscle: An Emerging Point of View on Hepatic Encephalopathy

**DOI:** 10.3390/jcm11030611

**Published:** 2022-01-26

**Authors:** Simone Di Cola, Silvia Nardelli, Lorenzo Ridola, Stefania Gioia, Oliviero Riggio, Manuela Merli

**Affiliations:** Department of Translational and Precision Medicine, Centre for the Diagnosis and Treatment of Portal Hypertension, “Sapienza” University of Rome, 00185 Rome, Italy; simone.dicola@uniroma1.it (S.D.C.); lorenzo.ridola@uniroma1.it (L.R.); stensgioia@hotmail.com (S.G.); oliviero.riggio@uniroma1.it (O.R.); manuela.merli@uniroma1.it (M.M.)

**Keywords:** cirrhosis, sarcopenia, myosteatosis, hepatic encephalopathy

## Abstract

In the last years the link between the presence of muscular alterations and hepatic encephalopathy (HE), both minimal and overt, has been deeply studied. The pathophysiological background supporting the relationship between muscle depletion, and HE is characterized by an imbalance between the capacity of muscle in ammonia metabolism and trafficking and the inability of the liver in removing ammonia through urea synthesis due to liver failure and/or the presence of porto-systemic shunts. This review will focus on the clinical burden, the physio pathological mechanisms understanding the liver muscle axis and principles of management of muscular alterations in cirrhosis.

## 1. Introduction

Hepatic encephalopathy (HE) is defined as a spectrum of neuro/psychiatric alterations caused by liver failure and/or porto-systemic shunts with different clinical conditions ranging from subclinical alterations to coma [1]. It’s one of the most frequent complication of liver cirrhosis affecting up to 30–40% of cirrhotic patients. It can be divided into overt hepatic encephalopathy (OHE), that is clinically evident and minimal hepatic encephalopathy (MHE), a condition characterized by subclinical alterations detectable only with psychometric tests or electroencephalography. More recently the term “covert HE” has been coined to combining MHE and Grade I of overt HE. The aim is to unify the terminology, make the diagnosis clearer and allow the design of more inclusive and uniform clinical studies. The term “covert” refers to a condition that is not unapparent, but also not overt. Covert HE, if investigated, can reach up to 80% of cirrhotic patients [1]. It is well known that HE worsens the prognosis of patients with cirrhosis and it is one of the main causes of hospitalization [2]; in particular MHE has been associated with falls [3,4] and car accidents [5,6,7], a reduction in quality of life and the socio-economic status and has a major impact on economic health costs [8,9,10]. 

Another common complication of liver cirrhosis is malnutrition; it correlates with the severity of liver disease and disease complications, including hepatic encephalopathy [11]. Sarcopenia, the generalized loss of muscle mass and function, is the major component of malnutrition [12]. Skeletal muscle is the main protein reserve in the body, it is maintained thanks to a continuous balance between protein synthesis and catabolism (proteostasis), and represents up to half of the entire protein turnover of the body [13]. Although it is a pathophysiological mechanism that correlates with the advancement of age (primitive sarcopenia), some chronic pathologies can accelerate this process (secondary sarcopenia) [13]. 

Sarcopenia is present between 30% and 70% of cirrhotic patients, with an increasing trend depending on the degree of liver disease [14]. Sarcopenia is a part of the frailty complex in cirrhotic patients, with a decreased reserve and resistance to stressors. Furthermore, it has been demonstrated that sarcopenia increases mortality in cirrhotic patients. In recently published metanalysis by Tantai et al. [15], authors conclude that sarcopenia was highly and independently associated with higher risk of mortality in patients with cirrhosis.

It has been suggested that considering muscle alterations in addition to prognostic scores improves the prediction of death in patients with cirrhosis [16]. Despite this, nutrition is often overlooked as nutritional assessment could be complex in cirrhotic patients [17]. Moreover, muscular alterations not only affect muscle mass but also its quality and function, making the picture more complex.

The methods for the diagnosis of sarcopenia are not uniform. This makes the overall evaluation of the studies difficult. They can be based on the different aspects of sarcopenia, that are the assessment of muscle mass, muscle performance and muscle strength. The gold standard for assessing sarcopenia is CT-scan, which allows to evaluate the muscle mass at the level of the L3 section [17].

The link between muscle alterations and liver disease is not well defined; it is multifactorial and includes hormonal alterations [18], hyperammonemia [19,20] and endotoxinemia [21]. Portal hypertension could play a role regardless of liver function in sarcopenia genesis [22], although this link needs to be better investigated. Therapies that act by reducing portal hypertension, such as transjugular intrahepatic portosystemic shunt, seem to improve muscle structure [23,24].

Among the etiological causes one of the most important roles is certainly given by hyperammonemia, which is probably the most evident expression of the close link that existing between liver and muscle. For this reason, cirrhotic patients should never be evaluated without considering the nutritional aspect and muscular alterations that are often present.

## 2. Muscle Alterations and Hepatic Encephalopathy in Liver Cirrhosis

During the natural history of chronic liver disease, muscle alterations may appear and these include muscle wasting, sarcopenia and myosteatosis. Sarcopenia is the most prevalent muscle abnormality, with a generalized reduction in muscle mass and function. It is defined as a muscle mass two standard deviations below the healthy young adult mean [25]. Myosteatosis is a fat infiltration of the skeletal muscle mass with an increased proportion of intramuscular and intermuscular fat that could impact muscle function and lead to a systemic inflammation [26].

The presence of myosteatosis and sarcopenia has been associated with a poor prognosis in patients with liver cirrhosis [27]. Moreover, these conditions are associated to several complications of liver cirrhosis, such as ascites [28], Spontaneous bacterial peritonitis (SBP) [29], variceal bleeding [30], hepatocellular carcinoma [31] and infections [32,33]. 

One of the closest and well-known relationship between muscle alterations and complications of liver disease is between sarcopenia and hepatic encephalopathy [34] (Table 1).

### 2.1. Sarcopenia and Cognitive Impairment

From as far back as 1964 it has been known that malnutrition could impact the prognosis of patients with liver cirrhosis in specific settings [35]. This link was confirmed by a large Italian multicentric prospective study conducted in 1996 [36]. Since that moment, several studies were conducted to investigate the link between malnutrition and liver complications. Among these, it was immediately clear that patients with malnutrition were at higher risk to develop cognitive impairment. 

Soros et al. in 2008 analyzed 223 cirrhotic patients’ muscle mass (assessed by bioelectrical impedance analysis): parameters of fat and fat-free mass were found to be similar in patients with and without HE [37].

Despite this, more recent studies demonstrated different results. Using handgrip strength (HGS) in 84 cirrhotic patients in 2011, Huisman et al. [28] found that muscle strength was an independent predictor of complications (including HE) after correcting for comorbidities, age and Child Pugh score. In a large cohort of patients (675 cirrhotic patients enrolled from 2000 to 2014) [38], sarcopenia was found to be associated with a higher risk of overt hepatic encephalopathy (OHE). In this study, sarcopenia was evaluated with CT scan, in particular cross-sectional areas were obtained from transverse CT images at the level of L3 of each patient and adjusted for height to calculate the Skeletal Muscle Index (SMI). In this study there was a strong correlation between sarcopenia and mortality, after adjusting for multiple confounding factors. 

More recently, a Japanese group retrospectively analyzed nearly 300 patients that have a HGS measurement [39]. They found that HGS was able to stratify patients at high risk to develop OHE. Despite the retrospective nature of this study, it demonstrated that a simple and economical measurement bedside of the patient can reliably discriminate patients at risk for this complication. 

In 2013 Merli et al. [40] enrolled 300 cirrhotic patients and at multivariate logistic regression analysis, muscle depletion, evaluated with BMI, mid-arm-muscle-circumference (MAMC), triceps skinfold-thickness (TSF) and HGS, was found as an independent risk factor for OHE during hospitalization. Moreover, this study was one of the first study that investigated the relationship between muscle depletion and minimal hepatic encephalopathy (MHE). MHE is a subclinical condition in which cognitive impairment isn’t detectable with physical examination, but only with psychometric tests [41,42], electrophysiological and other functional brain measures [43,44]. In this study [41] MHE was evaluated with psychometric tests and the reduction in muscle mass and muscle function were significantly associated not only with overt HE but also with MHE. Although the relationship between muscular alterations and MHE was directly researched for the first time in this study, in 2007 it has been demonstrated that patients with malnutrition (assessed by anthropometry and estimation of recent weight change) and patients with diabetes mellitus were at higher risk of cognitive impairment [45]. 

In a retrospective study conducted by Hanai et al. in 2017 [46], appendicular skeletal muscle mass (ASM) using bio-impedance analysis and HGS were performed to investigate the presence of sarcopenia. In this cohort of patients (120) sarcopenia was strongly associated with the presence of MHE. In addition to the retrospective and single-center nature of this study, another limit is that MHE was investigated with number connection test-A (NCT-A), number connection test-B (NCT-B), digit symbol test (DST) and block design test (BDT), a combination easier and quicker than the gold-standard PHES (psychometric hepatic encephalopathy score) [11].

However, all these results have been recently confirmed in a prospective study of 64 patients with liver cirrhosis [34]. The muscle assessment was investigated with CT-scan using Carey’s cut-off of the SMI for determination of sarcopenia [47]. Thirty-two patients (50%) had MHE at the time of enrollment, of whom 84% had sarcopenia; only 31% of patients without MHE had sarcopenia. In the multivariate analysis, only sarcopenia, myosteatosis and previous episodes of HE, were independently associated to the presence of MHE.

### 2.2. Myosteatosis and Cognitive Impairment

It is well known that the reduction in muscle mass is not the only muscle alteration that can be associated with chronic liver disease. The infiltration of muscle mass by intermuscular and intramuscular fat was first described in 1983 because of ageing [48] and metabolic abnormalities [49] and later defined as myosteatosis [50]. It is associated with poorer muscle strength and physical performance in older persons. As in sarcopenia, it has been demonstrated that this condition can appears also in younger people with chronic disease [51].

Montano-Loza et al. [27] have demonstrated that sarcopenia and myosteatosis increase the risk of mortality by 1.5-to twofold compared with patients without muscular abnormalities. Whereas these are very frequent alterations in cirrhotic patients, it’s important to investigate them. In this study, however, they have considered mainly patients with advanced liver cirrhosis (Child-Pugh B-C) and the percent of hepatocellular carcinoma was quite high. 

Few studies have analyzed the relationship between myosteatosis and HE. Bhanji et al. have studied a large cohort of cirrhotic patients with an available CT-scan in a retrospective analysis [38]. At multivariable regression analysis, myosteatosis was independently associated with a higher risk of HE. Patients with HE and myosteatosis had worse survival (15 ± 8 months), in comparison to those without these conditions (58 ± 14 months; *p* = 0.001) or with only HE or myosteatosis (31 ± 6 months; *p* = 0.02). Nardelli et al. investigated for the first time the link between myosteatosis and MHE [34], demonstrating that myosteatosis was strongly associated not only with OHE but also with MHE.

**Table 1:** Studies evaluating the relationship between muscle alterations and hepatic encephalopathy in cirrhosis.

First Author (Year)	Number of Patients	Methods to Identify Sarcopenia and/or Myosteatosis	Prevalence of Sarcopenia and/or Myosteatosis	Results
Merli et al. (2013) [40]	300 hospitalized cirrhotics	Anthropometric measurements (MAMC) and handgrip strenght (HGS)	48%	Overt HE in 30% with sarcopenia vs. 15% without sarcopenia (*p* = 0.003) Minimal HE in 49% with sarcopenia vs. 30% without sarcopenia (*p* = 0.001)
Hanai et al. (2017) [46]	120 cirrhotics	Bio-impedance Analysis (BIA), handgrip strenght	27%	Sarcopenia and serum branched-chain amino acids levels were associated with MHE in the multivariate analysis (*p* = 0.02 and *p* = 0.03 respectively).
Miwa et al. (2021) [39]	270 cirrhotics	Handgrip strength	38%	Multivariate analysis showed that reduced HGS was associated with a higher prevalence of CHE and higher risk for developing OHE
Nardelli et al. (2017) [52]	46 cirrhotics submitted to TIPS	CT scan to evaluate sarcopenia with Skeletal Muscle Index (SMI)	57%	Twenty-one patients (46%) developed overt HE after TIPS placement; all of these patients were sarcopenic. At multivariate analysis, only MELD score (*p* = 0.043) and sarcopenia (*p* < 0.001) were independently associated with the development of HE after TIPS placement.
Kalaitzakis et al. (2007) [45]	128 cirrhotic patients	BMI, weight loss, MAMC and triceps skinfold	40%	HE in 46% with malnutrition vs. 27% without malnutrition (*p* = 0.03)
Huisman et al. (2011) [30]	84 cirrhotic patients	Handgrip strength	67%	Increased complications in cirrhotic patients with lower muscle function, including HE (18% vs. 48%, *p* = 0.007)
Nardelli et al. (2019) [34]	64 cirrhotic patients	CT scan to evaluate sarcopenia and myosteatosis	Sarcopenia 58% Myosteatosis 38%	Both myosteatosis and sarcopenia were more frequent in patients who developed overt HE. On multivariate analysis, only sarcopenia (*p* = 0.005) and myosteatosis (*p* = 0.002) were independently associated to the development of overt HE.
Bhanji et al. (2018) [38]	675 cirrhotic patients	CT scan to evaluate sarcopenia and myosteatosis	Sarcopenia 36% Myosteatosis 52%	Both myosteatosis (70 vs. 45%, *p* < 0.001) and sarcopenia (53 vs. 32%, *p* < 0.001) were more frequent in patients with hepatic encephalopathy. By multivariable regression analysis, both myosteatosis and sarcopenia were associated with a higher risk of hepatic encephalopathy, independent of the MELD score.

## 3. Liver-Muscle Axis and Hyperammonemia: A Link to Explore

It has long been known that liver cirrhosis is a systemic disease which, especially in the advanced stages, affects different organs and systems. Although suspected for a long time [53], it has only recently become evident that the relationship between liver and muscle is very close, where one affects the other. Data collected until now show that both muscle synthesis and lysis can be altered in cirrhosis. 

Regarding the synthesis of muscle, several factors reduce the potential of the organism to synthesize muscle mass. First, cirrhotic patients are known to have a reduced calories intake; the presence of ascites can lead to early satiety due to increase in abdominal pressure. In turn sarcopenia contributes to fatigue and limits exercise tolerance, reduces performance status and activities of daily living; reduced physical activity obviously contributes to reduced anabolic stimulation [54], which is already altered in the patient with liver cirrhosis [55]. Notably, contrary to what is always claimed, the low-sodium diet could be counterproductive in cirrhotic patients with ascites, making food less palatable and therefore leading the patient to take less calories. Moreover, cirrhotic patients have different causes of malabsorption, such as reduced bile flow with malabsorption of fat-soluble vitamins and fats, pancreatic insufficiency in alcoholic related liver disease with alteration in absorption of fats, bacterial overgrowth due to impaired intestinal motility and portal hypertension [56]. Second, testosterone is an anabolic hormone that increases muscle mass by improving levels of insulin-like growth factor-1 (IGF-1), also called mechano-growth factor [57]. Through IGF-1, testosterone is able to activate mammalian target of rapamycin (mTOR), which is a crucial point in the activation of muscle synthesis. As is well know, cirrhotic patients have low testosterone levels [58]. Finally, leucine-enriched BCAAs have an important role in the synthesis of muscle mass [59]. All these mechanisms, through the stimulation of mTOR, lead to the activation of satellite muscle cells. These cells live in a state of quiescence and when stimulated allow the restoration of muscle mass [60].

On the other hand, proteolysis is upregulated in cirrhotic patients. Above all, cirrhosis is a hypermetabolic condition due to proinflammatory state. In this way, the organism uses gluconeogenesis to compensate for glycogen deficiencies, already altered in the cirrhotic, by consuming proteins and muscle mass. In this perspective, prolonged fasting should be avoided. Chronic inflammation induces autophagy by activating the ubiquitin-proteasome system [61]. Finally, a fundamental regulator of proteostasis is myostatin, a TGFB superfamily member that induces muscle loss. This regulation is due to the inhibition of the mammalian target of rapamycin complex 1 (mTORC1) [55].

Within this complex system of regulations, hyperammonemia has one of the most important roles. Ammonia is a compound of nitrogen and hydrogen, mostly a gut-derived toxin produced by bacterial metabolism of urea from proteins that are consumed in the human diet (urease-producing bacterial organisms). With the progression of liver disease, the microbiome enters a dysbiosis state, leading to greater inflammation and cholestasis. So, the composition of gut microbiota becomes altered and plays an important role in the pathogenesis of HE [62]. Moreover ammonia is generated from the continuous amino acid catabolism and purine turnover. From the catabolism inside the enterocytes and from the bacterial production, ammonia enters the portal system and then reaches the liver. Within the liver, ammonia passes through two filter systems, periportal hepatocytes and perivenous hepatocytes. Ammonia is used in the first system as a substrate of the urea cycle. However, in the perivenous hepatocytes there is a strong expression of the enzyme glutamine synthetase which removes the remaining part of toxin to prevent it from entering the systemic circulation [63]. When the liver is damaged and subverted by chronic injury and/or when collateral circles are established that pass the liver filter, ammonia passes directly into the systemic circulation and produces its cytotoxic effects at the level of the central nervous system. At this point the mechanism leading to hepatic encephalopathy is established. What is the role of the muscle within this liver-brain link? Skeletal muscle also expresses glutamine synthetase, although its activity is very low. However, considering the entire muscle extension, it is possible that the muscle is a good buffer system to dispose of excess circulating ammonia [64]. From this, if the skeletal muscle is reduced it has a lower ability to absorb circulating ammonia and therefore the risk of hepatic encephalopathy increases. Merli et al. have demonstrated that venous blood ammonia levels were significantly higher in patients with muscle depletion and in patients with a decreased muscle strength [40]. 

In this context, hyperammonemia has a direct negative effect on muscle turn-over. Its action is polyhedral and acts both on synthesis and on muscle lysis. Moreover, hyperammonemia induces a cellular stress response and mimes cell responses activated by amino acid deficiency. In particular, it inhibits the translation of mRNA and protein synthesis into skeletal muscle through activation of general control non-depressed 2 (GCN2) and inhibition of mTORC1 with unknown mechanisms [65]. In addition, the disturbance of the tricarboxylic acid cycle, linked to the loss of alpha-KG (necessary for the conversion of ammonia into glutamate) leads to loss of ATP, mitochondrial dysfunction, reduction of contractile function and finally to sarcopenia. Ammonia also can potentially cause post-translational modifications, including protein nitration and oxidative stress-induced carbonylation of contractile proteins with impaired actomyosin interactions. That is why ammonia-mediated nitration is a potential molecular mechanism of impaired contractile function [20].

It has also been widely demonstrated that hyperammonemia leads to increased activation of myostatin in cirrhotic patients [66], inhibiting protein synthesis. Nishikawa et al. [67] have demonstrated that higher levels of myostatin is associated with hyperammonemia and muscle loss in cirrhotic patients; moreover, patients with increased myostatin had worse prognosis, suggesting the importance of muscle in the prognostic overview of the patient with liver cirrhosis. This concept echoes the above-mentioned idea of considering sarcopenia in the predictive mortality model for cirrhotic patients in liver transplant evaluation [16].

Finally, it has been demonstrated that hyperammonemia increases autophagy in cirrhotic patients with unclear mechanisms [19].

Regarding myosteatosis, the physiopathological association with hyperammonemia and HE is more complex and partially unknown. Myosteatosis seems to derive from a complex mechanism involving the metabolism of fatty acids and glycogen; the pivotal point of this process is mediated by the proinflammatory state that is present and that leads to muscle depletion [26]. Nardelli et al. have demonstrated the association between myosteatosis and HE, hypothezing that fat infiltration, by reducing the fat-free mass, may contribute to the reduction of leads to partial loss of function of glutamate synthetase, expressed in the muscle cell. The significant correlation between ammonia, SMI, and muscle attenuation seems to support this hypothesis [34]. 

In summary, the set of these mechanisms activates a vicious circle in which cirrhosis induces muscular depletion with multiple mechanisms; the muscular deficiency on the other hand reduces the capacities of absorption of circulating ammonia. Hyperammonemia in turn alters proteostasis and induces further muscle loss. It is now known that muscle depletion reduces survival in cirrhotic patients. The biochemical link of the liver-muscle axis is probably more complex than said and given its prognostic importance it must be further investigated to find therapy targets that can block this vicious circle.

## 4. Clinical and Therapeutic Management

Although it has long been known that muscular alterations impaired survival and quality of life of patients with cirrhosis, definite and effective therapeutic approaches are not yet available. Several therapeutic alternatives have been explored, but extensive evidence on the effectiveness of these approaches is still lacking. Surely the cirrhotic patient at risk of malnutrition must be managed in a multidisciplinary way and carefully followed up also from a nutritional point of view given the strong bidirectional link between muscle and liver. Thus, the management of malnutrition and muscular alterations is also important for the prevention of complications of liver disease, especially HE. If the skeletal muscular system has a good function of disposing of excess circulating ammonia, acting on muscle mass and function could be a target therapy for HE. To date, different therapeutic approaches for the management of muscular alterations have been investigated (Table 2).

It has been proven that moderate physical activity increases muscle mass and function in patients with cirrhosis [68,69]. However, it is not known how much this impacts on survival and long-term complications. Another important approach consists of increase calorie intake and prefer small and frequent meals with night snacks before going to sleep as suggested by international guidelines [12] to compensate the condition of hypermetabolism frequent in cirrhotic patients [70].

Therapies aimed at managing either HE or muscle mass could play a role in both situations, having muscle and HE a very close bond. By improving muscle mass, it is possible to increase its ability to dispose of circulating levels of ammonia, due to the pathophysiological mechanisms described above. Different therapeutic possibilities have been proposed, such as testosterone, IGF1 [71] and inhibitors of myostatin (follistatin) [72,73]. However, there are few evidence, especially in human models. The use of testosterone has been more studied. Fifteen studies were analyzed in a recent systematic review [74], nine of which were interventional. Although both observational and interventional studies have shown that a low level of testosterone in cirrhotic correlates with sarcopenia, disease decompensation and death, testosterone supplementation cannot improve survival, the risk of decompensation and hepatic encephalopathy, despite the increase in muscle mass [74].

The cataplerosis (loss of critical TCA cycle intermediate and alfa-KG) that is present in cirrhotic patients, could be reversed by oral supplementation of BCAA. They are deaminated to provide carbon skeleton for TCA cycle. Lower levels of BCAA in plasma and muscle have been prompted by hyperammonemia [75,76]. Miwa et al. have demonstrated that low serum BCAA levels can be the predictor of MHE in patients with cirrhosis [39]. Although the integration of BCAA would appear to increase ammonia plasma levels in the short term by increasing glutamine synthesis, prolonged administration would lead to the reduction of hyperammonemia [77]. Among these, isoleucine and its active metabolite β-hydroxy-β-methylbutyrate (HMB) were widely studied for the anti-catabolic effects in skeletal muscle. Lattanzi et al. [78] have demonstrated that adequate dietary intake and physical activity counseling alongside HMB supplementation for 12 weeks improve muscle performance without modification in cognitive status evaluated by the PHES test; notably, none of the 24 patients had cognitive alterations at the baseline.

Ammonia-lowering therapy could be useful in improving muscle mass, by inhibiting autophagy and the expression of myostatin. It has been demonstrated on animal models that combined therapy of LOLA (L-ornithine L-aspartate) plus rifaximin increases strength and muscle mass [79]. In an old study in cirrhotic patients the use of LOLA compared to placebo improved protein synthesis rates measured directly in muscle biopsies [80]. The action of LOLA in this context is manifold. It acts both on the muscle and on the liver. In the periportal hepatocytes LOLA induces the conversion of ammonia into urea. It also ensures the production of glutamate that is exploited by GS to convert ammonia to glutamine in the muscle [81]. Finally, it seems to have anti-oxidant power on the liver and hepatic-protective functions, thus reducing liver dysfunction and hyperammonemia [82].

Finally, TIPS involves substantial changes in the setting of hyperammonemia and muscle state. TIPS is known to worsen hyperammonemia by increasing the shunt of portal blood that bypasses the liver. Sarcopenia has been shown to be a negative prognostic factor for the risk of MHE and OHE after TIPS placement [52]. Probably a poor muscle reserve reduces the muscle’s ability to buffer the hyperammonemia. On the other hand, it seems that the positioning of TIPS can improve muscle mass despite the increase of ammonemia [24]. This is probably linked to the reduction of portal hypertension which plays an independent role in the genesis of muscular alterations. It has been shown that even in the absence of significant liver alterations the presence of portal hypertension may be associated with the development of sarcopenia [24].

**Table 2:** Studies evaluating the different interventions tried for improving sarcopenia in cirrhosis.

First Author (Year)	Number of Patients	Type of Treatment and Duration	Method to Explore Muscle Depletion or Nutritional Impairment	Results
Muto et al. (2005) [77]	646 with decompensat ed cirrhosis	BCAA orally (12 g/day) Vs diet therapy for 2 years	albumin concentration and health-related quality of life (QOL) measured by Short Form-36 questionnaire	The incidence of death and event free survival significantly decreased in the BCAA group (*p*: 0.015). Regarding hyperammonemia, exhibited lower mean blood ammonia levels than the diet group, with no statistical significance.
Lattanzi et al. (2021) [78]	24 cirrhotics	HMB orally 3 gr/die vs. placebo for 12 weeks	anthropometry, electrical bioimpedance analysis (BIA), quadriceps ultrasound, physical performance battery, Liver Frailty Index (LFI), and cognitive tests	Improving in muscle performance without modification in cognitive status evaluated by the PHES test. Nb: none of the 24 patients had cognitive alterations at the baseline
Roman et al. (2016) [68]	23 cirrhotics	14 patients randomized to an exercise program vs. 9 patients to relaxation program	Anthropometry, Dual-energy X-ray absorptiometry (DEXA), Time Up & Go (TUG)	The exercise group shows a decrease in fat body mass (*p* = 0.003), increase in lean body mass (*p* = 0.01), lean appendicular mass (*p* = 0.03) and lean leg mass (*p*= 0.02). TUG decreased at the end of the study (*p* = 0.02).
Gioia et al. (2019) [24]	27 cirrhotic patients submitted to TIPS	TIPS placement	Skeletal muscle index at CT scan lumbar 3	SMI significantly improved after TIPS placement (*p* = 0.0001). Patients with improved SMI had reduced number of episodes of OHE and prevalence of MHE (*p* = 0.0001)
Deng et al. (2021) [74]	580 cirrhotic patients	Testosterone	Various	Testosterone supplementation improved appendicular mass and bone mineral density with no results in terms of liver decompensation and death

## 5. Conclusions and Further Prospectives

The concept that liver and muscle are two different entities has long since passed. The interaction between muscle and liver are numerous and the alterations of one can involve considerable modifications in the other. The task of the clinician is therefore to consider the cirrhotic patient as a whole in his alterations, including the nutritional and muscular state. However, studies demonstrating these links are often small and retrospective; information is often fragmented and does not provide a complete picture. It is therefore necessary in the future to design extensive and prospective studies with epidemiological descriptions and natural history of cirrhotic patients with muscular alterations.

Moreover, the presence of muscle alterations is establishing itself as one of the heaviest factors capable of influencing the prognosis of the cirrhotic patient, identifying a subgroup of individuals at truly “high risk”. It would be important that future studies also focus on the possibility of developing prognostic scores capable of considering this variable, together with other emerging ones such as the presence of spontaneous shunts, to identify this subgroup of patients deserving of more intense management.

It will also be necessary to better define the role of some of the most promising therapies and the weight of dietary and lifestyle changes in this group of patients. It is necessary to define better the role that ammonia-lowering therapies can have in improving the altered proteostasis of cirrhotic patients. Finally, it is likely that gender may have a different effect on the muscle-liver axis and the natural history of cirrhotic patients with muscular alterations; however, little effort has been made on this aspect; gender studies must therefore be developed in the future. 

## References

[1] Vilstrup H., Amodio P., Bajaj J., Cordoba J., Ferenci P., Mullen K.D., Weissenborn K., Wong P. (2014). Hepatic encephalopathy in chronic liver disease: 2014 Practice Guideline by the American Association for the Study of Liver Diseases and the European Association for the Study of the Liver. Hepatology.

[2] Scaglione S.J., Metcalfe L., Kliethermes S., Vasilyev I., Tsang R., Caines A., Mumtaz S., Goyal V., Khalid A., Shoham D. (2017). Early Hospital Readmissions and Mortality in Patients with Decompensated Cirrhosis Enrolled in a Large National Health Insurance Administrative Database. J. Clin. Gastroenterol..

[3] Roman E., Córdoba J., Torrens M., Torras X., Villanueva C., Vargas V., Guarner C., Soriano G. (2011). Minimal Hepatic Encephalopathy Is Associated with Falls. Am. J. Gastroenterol..

[4] Nardelli S., Gioia S., Ridola L., Carlin M., Cioffi A.D., Merli M., Riggio O., Spagnoli A. (2021). Risk of falls in patients with cirrhosis evaluated by timed up and go test: Does muscle or brain matter more?. Dig. Liver Dis..

[5] Kircheis G., Knoche A., Hilger N., Manhart F., Schnitzler A., Schulze H., Häussinger D. (2009). Hepatic Encephalopathy and Fitness to Drive. Gastroenterology.

[6] Bajaj J.S., Saeian K., Hafeezullah M., Hoffmann R.G., Hammeke T.A. (2008). Patients with Minimal Hepatic Encephalopathy Have Poor Insight into Their Driving Skills. Clin. Gastroenterol. Hepatol..

[7] Lauridsen M.M., Thacker L.R., White M.B., Unser A., Sterling R.K., Stravitz R.T., Matherly S., Puri P., Sanyal A.J., Gavis E.A. (2016). In patients with cirrhosis, driving simulator performance is associated with real-life driving. Clin. Gastroenterol. Hepatol..

[8] Ridola L., Cardinale V., Riggio O. (2018). The burden of minimal hepatic encephalopathy: From diagnosis to therapeutic strategies. Ann. Gastroenterol..

[9] Stepanova M., Mishra A., Venkatesan C., Younossi Z.M. (2012). In-Hospital Mortality and Economic Burden Associated with Hepatic Encephalopathy in the United States From 2005 to 2009. Clin. Gastroenterol. Hepatol..

[10] Roggeri A., Roggeri D.P., Rossi E., Cinconze E., Gasbarrini A., Preti P.M., De Rosa M. (2015). Overt hepatic encephalopathy in Italy: Clinical outcomes and healthcare costs. Hepatic Med. Évid. Res..

[11] Montagnese S., Russo F.P., Amodio P., Burra P., Gasbarrini A., Loguercio C., Marchesini G., Merli M., Ponziani F.R., Riggio O. (2019). Hepatic encephalopathy 2018: A clinical practice guideline by the Italian Association for the Study of the Liver (AISF). Dig. Liver Dis..

[12] European Association for the Study of the Liver (2019). EASL Clinical Practice Guidelines on nutrition in chronic liver disease 2019. J. Hepatol..

[13] Rennie M.J., Tipton K.D. (2000). Protein and amino acid metabolism during and after exercise and the effects of nutrition. Annu. Rev. Nutr..

[14] Kim G., Kang S.H., Kim M.Y., Baik S.K. (2017). Prognostic value of sarcopenia in patients with liver cirrhosis: A systematic review and meta-analysis. PLoS ONE.

[15] Tantai X., Liu Y., Yeo Y.H., Praktiknjo M., Mauro E., Hamaguchi Y., Engelmann C., Zhang P., Jeong J.Y., van Vugt J.L.A. (2021). Effect of sarcopenia on survival in patients with cirrhosis: A systematic review and meta-analysis. J. Hepatol..

[16] Lattanzi B., Nardelli S., Pigliacelli A., Di Cola S., Farcomeni A., D’ambrosi D., Gioia S., Corradini S.G., Lucidi C., Mennini G. (2019). The additive value of sarcopenia, myosteatosis and hepatic encephalopathy in the predictivity of model for end-stage liver disease. Dig. Liver Dis..

[17] Montano-Loza A.J. (2014). Clinical relevance of sarcopenia in patients with cirrhosis. World J. Gastroenterol..

[18] Jindal A., Jagdish R.K. (2019). Sarcopenia: Ammonia metabolism and hepatic encephalopathy. Clin. Mol. Hepatol..

[19] Qiu J., Tsien C., Thapalaya S., Narayanan A., Weihl C.C., Ching J.K., Eghtesad B., Singh K., Fu X., Dubyak G. (2012). Hyperammonemia-mediated autophagy in skeletal muscle contributes to sarcopenia of cirrhosis. Am. J. Physiol. Metab..

[20] McDaniel J.G., Davuluri G., Hill E.A., Moyer M., Runkana A., Prayson R., Van Lunteren E., Dasarathy S. (2016). Hyperammonemia results in reduced muscle function independent of muscle mass. Am. J. Physiol. Gastrointest. Liver Physiol..

[21] Kovarik M., Muthny T., Sispera L., Holecek M. (2012). The dose-dependent effects of endotoxin on protein metabolism in two types of rat skeletal muscle. J. Physiol. Biochem..

[22] Lattanzi B., Gioia S., Di Cola S., D’Ambrosio D., Nardelli S., Tavano D., Farcomeni A., Merli M., Riggio O. (2019). Prevalence and impact of sarcopenia in non-cirrhotic portal hypertension. Liver Int..

[23] Plauth M., Schütz T., Buckendahl D.P., Kreymann G., Pirlich M., Grüngreiff S., Romaniuk P., Ertl S., Weiß M.-L., Lochs H. (2004). Weight gain after transjugular intrahepatic portosystemic shunt is associated with improvement in body composition in malnourished patients with cirrhosis and hypermetabolism. J. Hepatol..

[24] Gioia S., Merli M., Nardelli S., Lattanzi B., Pitocchi F., Ridola L., Riggio O. (2019). The modification of quantity and quality of muscle mass improves the cognitive impairment after TIPS. Liver Int..

[25] Baumgartner R.N., Koehler K.M., Gallagher D., Romero L., Heymsfield S.B., Ross R.R., Garry P.J., Lindeman R.D. (1998). Epidemiology of Sarcopenia among the Elderly in New Mexico. Am. J. Epidemiol..

[26] Miljkovic I., Vella C.A., Allison M. (2021). Computed Tomography-Derived Myosteatosis and Metabolic Disorders. Diabetes Metab. J..

[27] Montano-Loza A.J., Angulo P., Meza-Junco J., Prado C.M.M., Sawyer M.B., Beaumont C., Esfandiari N., Ma M., Baracos V.E. (2015). Sarcopenic obesity and myosteatosis are associated with higher mortality in patients with cirrhosis. J. Cachex-Sarcopenia Muscle.

[28] Huisman E.J., Trip E.J., Siersema P.D., Van Hoek B., Van Erpecum K.J. (2011). Protein energy malnutrition predicts complications in liver cirrhosis. Eur. J. Gastroenterol. Hepatol..

[29] Zeng X., Shi Z., Yu J., Wang L., Luo Y., Jin S., Zhang L., Tan W., Shi P., Yu H. (2021). Sarcopenia as a prognostic predictor of liver cirrhosis: A multicentre study in China. J. Cachex-Sarcopenia Muscle.

[30] Møller S., Bendtsen F., Christensen E., Henriksen J.H. (1994). Prognostic variables in patients with cirrhosis and oesophageal varices without prior bleeding. J. Hepatol..

[31] Feng Z., Zhao H., Jiang Y., He Z., Sun X., Rong P., Wang W. (2020). Sarcopenia associates with increased risk of hepatocellular carcinoma among male patients with cirrhosis. Clin. Nutr..

[32] Merli M., Lucidi C., Giannelli V., Giusto M., Riggio O., Falcone M., Ridola L., Attili A.F., Venditti M. (2010). Cirrhotic Patients Are at Risk for Health Care–Associated Bacterial Infections. Clin. Gastroenterol. Hepatol..

[33] Lucidi C., Lattanzi B., Di Gregorio V., Incicco S., D’Ambrosio D., Venditti M., Riggio O., Merli M. (2018). A low muscle mass increases mortality in compensated cirrhotic patients with sepsis. Liver Int..

[34] Nardelli S., Lattanzi B., Merli M., Farcomeni A., Gioia S., Ridola L., Riggio O. (2019). Muscle Alterations Are Associated With Minimal and Overt Hepatic Encephalopathy in Patients With Liver Cirrhosis. Hepatology.

[35] Child C.G., Turcotte J.G., Child C.G. (1964). Surgery and portal hypertension. The Liver and Portal Hypertension.

[36] Merli M., Riggio O., Dally L. (1996). Does malnutrition affect survival in cirrhosis? PINC (Policentrica Italiana Nutrizione Cirrosi). Hepatology.

[37] Sörös P., Böttcher J., Weissenborn K., Bengtsson M., Jalan R., Björnsson E. (2008). Malnutrition and hypermetabolism are not risk factors for the presence of hepatic encephalopathy: A cross-sectional study. J. Gastroenterol. Hepatol..

[38] Bhanji R.A., Moctezuma-Velazquez C., Duarte-Rojo A., Ebadi M., Ghosh S., Rose C., Montano-Loza A.J. (2018). Myosteatosis and sarcopenia are associated with hepatic encephalopathy in patients with cirrhosis. Hepatol. Int..

[39] Miwa T., Hanai T., Nishimura K., Maeda T., Ogiso Y., Imai K., Suetsugu A., Takai K., Shiraki M., Shimizu M. (2021). Handgrip strength stratifies the risk of covert and overt hepatic encephalopathy in patients with cirrhosis. J. Parenter. Enter. Nutr..

[40] Merli M., Giusto M., Lucidi C., Giannelli V., Pentassuglio I., Di Gregorio V., Lattanzi B., Riggio O. (2012). Muscle depletion increases the risk of overt and minimal hepatic encephalopathy: Results of a prospective study. Metab. Brain Dis..

[41] Amodio P., Del Piccolo F., Marchetti P., Angeli P., Iemmolo R., Caregaro L., Merkel C., Gerunda G., Gatta A. (1999). Clinical features and survival of cirrhotic patients with subclinical cognitive alterations detected by the number connection test and computerized psychometric tests. Hepatology.

[42] Weissenborn K., Giewekemeyer K., Heidenreich S., Bokemeyer M., Berding G., Ahl B. (2005). Attention, memory, and cognitive function in hepatic encephalopathy. Metab. Brain Dis..

[43] McCrea M., Cordoba J., Vessey G., Blei A.T., Randolph C. (1996). Neuropsychological Characterization and Detection of Subclinical Hepatic Encephalopathy. Arch. Neurol..

[44] Amodio P., Montagnese S., Gatta A., Morgan M.Y. (2004). Characteristics of Minimal Hepatic Encephalopathy. Metab. Brain Dis..

[45] Kalaitzakis E., Olsson R., Henfridsson P., Hugosson I., Bengtsson M., Jalan R., Björnsson E. (2007). Malnutrition and diabetes mellitus are related to hepatic encephalopathy in patients with liver cirrhosis. Liver Int..

[46] Hanai T., Shiraki M., Watanabe S., Kochi T., Imai K., Suetsugu A., Takai K., Moriwaki H., Shimizu M. (2017). Sarcopenia predicts minimal hepatic encephalopathy in patients with liver cirrhosis. Hepatol. Res..

[47] Carey E.J., Lai J.C., Wang C.W., Dasarathy S., Lobach I., Montano-Loza A.J., Dunn M.A. (2017). A multicenter study to define sarcopenia in patients with end-stage liver disease. Liver Transplant..

[48] Borkan G.A., Hults B.D.E., Gerzof S.G., Robbins A.H., Silbert C.K. (1983). Age Changes in Body Composition Revealed by Computed Tomography. J. Gerontol..

[49] Petersen K.F., Befroy D., Dufour S., Dziura J., Ariyan C., Rothman D.L., DiPietro L., Cline G.W., Shulman G.I. (2003). Mitochondrial Dysfunction in the Elderly: Possible Role in Insulin Resistance. Science.

[50] Taaffe D.R., Henwood T.R., Nalls M.A., Walker D.G., Lang T.F., Harris T.B. (2009). Alterations in muscle attenuation following detraining and retraining in resistance-trained older adults. Gerontology.

[51] Goodpaster B.H., Carlson C.L., Visser M., Kelley D.E., Scherzinger A., Harris T.B., Stamm E., Newman A.B. (2001). Attenuation of skeletal muscle and strength in the elderly: The Health ABC Study. J. Appl. Physiol. (1985).

[52] Nardelli S., Lattanzi B., Torrisi S., Greco F., Farcomeni A., Gioia S., Merli M., Riggio O. (2017). Sarcopenia Is Risk Factor for Development of Hepatic Encephalopathy After Transjugular Intrahepatic Portosystemic Shunt Placement. Clin. Gastroenterol. Hepatol..

[53] Lockwood A.H., McDonald J.M., Reiman R.E., Gelbard A.S., Laughlin J.S., Duffy T.E., Plum F. (1979). The dynamics of ammonia metabolism in man. Effects of liver disease and hyperammonemia. J. Clin. Investig..

[54] Hayashi F., Matsumoto Y., Momoki C., Yuikawa M., Okada G., Hamakawa E., Kawamura E., Hagihara A., Toyama M., Fujii H. (2013). Physical inactivity and insufficient dietary intake are associated with the frequency of sarcopenia in patients with compensated viral liver cirrhosis. Hepatol. Res..

[55] Dasarathy S., Hatzoglou M. (2018). Hyperammonemia and proteostasis in cirrhosis. Curr. Opin. Clin. Nutr. Metab. Care.

[56] Romiti A., Merli M., Martorano M., Parrilli G., Martino F., Riggio O., Truscelli A., Capocaccia L., Budillon G. (1990). Malabsorption and nutritional abnormalities in patients with liver cirrhosis. Ital. J. Gastroenterol..

[57] Sinclair M., Grossmann M., Hoermann R., Angus P.W., Gow P.J. (2016). Testosterone therapy increases muscle mass in men with cirrhosis and low testosterone: A randomised controlled trial. J. Hepatol..

[58] Handelsman D.J., Strasser S., McDonald J.A., Conway A.J., McCaughan G.W. (1995). Hypothalamic-pituitary-testicular function in end-stage non-alcoholic liver disease before and after liver transplantation. Clin. Endocrinol..

[59] Fuchs C.J., Hermans W.J.H., Holwerda A., Smeets J., Senden J.M., Van Kranenburg J., Gijsen A.P., Wodzig W.K.H.W., Schierbeek H., Verdijk L. (2019). Branched-chain amino acid and branched-chain ketoacid ingestion increases muscle protein synthesis rates in vivo in older adults: A double-blind, randomized trial. Am. J. Clin. Nutr..

[60] Garikipati D.K., Rodgers B.D. (2012). Myostatin inhibits myosatellite cell proliferation and consequently activates differentiation: Evidence for endocrine-regulated transcript processing. J. Endocrinol..

[61] Beyer I., Mets T., Bautmans I. (2012). Chronic low-grade inflammation and age-related sarcopenia. Curr. Opin. Clin. Nutr. Metab. Care.

[62] Elwir S., Rahimi R.S. (2017). Hepatic Encephalopathy: An Update on the Pathophysiology and Therapeutic Options. J. Clin. Transl. Hepatol..

[63] Kaestner K.H. (2009). In the Zone: How a Hepatocyte Knows Where It Is. Gastroenterology.

[64] Desjardins P., Rao K.R., Michalak A., Rose C., Butterworth R.F. (1999). Effect of portacaval anastomosis on glutamine synthetase protein and gene expression in brain, liver and skeletal muscle. Metab. Brain Dis..

[65] Anda S., Zach R., Grallert B. (2017). Activation of Gcn2 in response to different stresses. PLoS ONE.

[66] Dasarathy S.J. (2017). Myostatin and beyond in cirrhosis: All roads lead to sarcopenia. Cachexia Sarcopenia Muscle.

[67] Nishikawa H., Enomoto H., Ishii A., Iwata Y., Miyamoto Y., Ishii N., Yuri Y., Hasegawa K., Nakano C., Nishimura T. (2017). Elevated serum myostatin level is associated with worse survival in patients with liver cirrhosis. J. Cachex-Sarcopenia Muscle.

[68] Román E., García-Galcerán C., Torrades T., Herrera S., Marín A., Doñate M., Alvarado-Tapias E., Malouf J., Nácher L., Serra-Grima R. (2016). Effects of an Exercise Programme on Functional Capacity, Body Composition and Risk of Falls in Patients with Cirrhosis: A Randomized Clinical Trial. PLoS ONE.

[69] Brustia R., Savier E., Scatton O. (2018). Physical exercise in cirrhotic patients: Towards prehabilitation on waiting list for liver transplantation. A systematic review and meta-analysis. Clin. Res. Hepatol. Gastroenterol..

[70] Okuda H., Shiratori K. (2007). Long-term nutritional assessment and quality of life in patients with cirrhosis taking a late evening snack. J. Gastroenterol..

[71] Picardi A., de Oliveira A.C., Muguerza B., Tosar A., Quiroga J., Castilla-Cortazar I., Santidrián S., Prieto J. (1997). Low doses of insulin-like growth factor-I improve nitrogen retention and food efficiency in rats with early cirrhosis. J. Hepatol..

[72] Dasarathy S., McCullough A.J., Muc S., Schneyer A., Bennett C.D., Dodig M., Kalhan S. (2011). Sarcopenia associated with portosystemic shunting is reversed by follistatin. J. Hepatol..

[73] Chen P.R., Lee K. (2016). Inhibitors of myostatin as methods of enhancing muscle growth and development. J. Anim. Sci..

[74] Deng N., Mallepally N., Peng F.B., Kanji A., Marcelli M., Hernaez R. (2021). Serum testosterone levels and testosterone supplementation in cirrhosis: A systematic review. Liver Int..

[75] Leweling H., Breitkreutz R., Behne F., Staedt U., Striebel J.-P., Holm E. (1996). Hyperammonemia-induced depletion of glutamate and branched-chain amino acids in muscle and plasma. J. Hepatol..

[76] Kitajima Y., Takahashi H., Akiyama T., Murayama K., Iwane S., Kuwashiro T., Tanaka K., Kawazoe S., Ono N., Eguchi T. (2017). Supplementation with branched-chain amino acids ameliorates hypoalbuminemia, prevents sarcopenia, and reduces fat accumulation in the skeletal muscles of patients with liver cirrhosis. J. Gastroenterol..

[77] Muto Y., Sato S., Watanabe A., Moriwaki H., Suzuki K., Kato A., Kato M., Nakamura T., Higuchi K., Nishiguchi S. (2005). Effects of Oral Branched-Chain Amino Acid Granules on Event-Free Survival in Patients With Liver Cirrhosis. Clin. Gastroenterol. Hepatol..

[78] Lattanzi B., Bruni A., Di Cola S., Molfino A., De Santis A., Muscaritoli M., Merli M. (2021). The Effects of 12-Week Beta-Hydroxy-Beta-Methylbutyrate Supplementation in Patients with Liver Cirrhosis: Results from a Randomized Controlled Single-Blind Pilot Study. Nutrients.

[79] Kumar A., Davuluri G., DeSilva R.N., Engelen M.P., Have G.A.T., Prayson R., Deutz N.E., Dasarathy S. (2017). Ammonia lowering reverses sarcopenia of cirrhosis by restoring skeletal muscle proteostasis. Hepatology.

[80] Reynolds N., Downie S., Smith K., Kircheis G., Rennie M. (1999). Treatment with L-ornithine L-aspartate (LOLA) infusion restores muscle protein synthesis responsiveness to feeding in patients with cirrhosis. J. Hepatol..

[81] Butterworth R.F. (2019). L-Ornithine L-Aspartate for the Treatment of Sarcopenia in Chronic Liver Disease: The Taming of a Vicious Cycle. Can. J. Gastroenterol. Hepatol..

[82] Butterworth R.F., Gruengreiff K. (2018). L-ornithine L-aspartate (LOLA) for the treatment of hepatic encephalopathy in cirrhosis: Evidence for novel hepatoprotective mechanisms. J. Liver Clin..

